# The complete mitochondrial genome of *Acanthurus achilles* (Acanthuriformes, Acanthuridae)

**DOI:** 10.1080/23802359.2020.1719921

**Published:** 2020-02-02

**Authors:** Tao Li, Yukai Yang, Xiaolin Huang, Heizhao Lin, Wei Yu, Zhong Huang, Xidong Mu

**Affiliations:** a Guangdong Provincial Key Laboratory of Fishery Ecology and Environment South China Sea Fisheries Research Institute, Guangzhou, China;; b Shenzhen Base of South China Sea Fisheries Research Institute, Chinese Academy of Fishery Sciences, Shenzhen, China;; c Pearl River Fisheries Research Institute, Chinese Academy of Fishery Sciences Key Laboratory of Recreational Fisheries, Ministry of Agriculture and Rural Areas, Guangzhou, China

**Keywords:** *Acanthurus Achilles*, Acanthuridae, mitochondrial genome

## Abstract

*Acanthurus achilles* is one of most important genera of Acanthuridae. However, the systemically classification and taxonomic studies have so far been limited. In this study, we report the complete mitochondrial genome sequence of *A*. *achilles*. The mitogenome has 16,537 base pairs (55.7% A + T content) and made up of total of 37 genes (13 protein-coding, 22 transfer RNAs and 2 ribosomal RNAs), and a putative control region. This study will provide useful genetic information for future phylogenetic and taxonomic classification of Acanthuridae.

*Acanthurus achilles* belongs to the Family Acanthuridae and the Order Acanthuriformes, the *A. achilles* has a striking black body with red and white accents. This attractive fish is considered a nervous swimmer as it frequently paces back and forth (Randall 1956).

There is no report of the complete genome of this species *A. achilles*, which was developed in Shenzhen, Guangdong Province, Republic of China (N22°37′34″, E114°41′06″) in October 2019. Therefore, it is very important to characterize the complete mitogenome of this species, which can be utilized in research on taxonomic resolution, population genetic structure and phylogeography, and phylogenetic relationship. Total DNA was extracted from muscle following TIANamp Marine Animals DNA Kit (Tiangen, China), and NOVOPlasty software was used to assemble the mitogenomes, the mistake parameter was set by default (Dierckxsens et al. 2017). The samples were stored in –80 °C in Key Lab of South China Sea Fishery Resources Exploitation and Utilization, Ministry of Agriculture and Rural Affairs, South China Sea Fisheries Research Institute, Chinese Academy of Fishery Sciences, Guangzhou, China. Number is AA-1.

In this study, we obtained the complete mitochondrial genome of the *A. achilles*. Its mitochondrial genome has been deposited in the GenBank under accession number MN872231. For a better understanding of genetic status and the evolutionary study, we focused on the genetic information contained in the complete mitochondrial genomes of the fish.

The complete mitogenome of the *A. achilles* was 16,537 bp in length. The genomic organization was identical to those of typical vertebrate mitochondrial genomes, including two rRNA genes, 13 protein-coding genes, 22 tRNA genes, a light-strand replication origin (OL), and a putative control region (CR). The overall base composition was 29.4% of A, 26.3% of T, 28.5% of C, and 15.8% of G with a slight A + T bias (55.7%) like other vertebrate mitochondrial genomes. The features mentioned above were accordant with typical Acanthuridae fish mitogenome.

*A. achilles* had two non-coding regions, the L-strand replication origin region (36 bp) locating between tRNA-Asn and tRNA-Cys, and the control region (759 bp) locating within the tRNA-Pro and tRNA-Phe. Except for eight tRNA (tRNA-Ser, tRNA-Pro, tRNA-Glu, tRNA-Tyr, tRNA-Cys, tRNA-Asn, tRNA-Ala, and tRNA-Gln) and the *ND6* gene were encoded on the L-strand, the others were encoded on the H-strand. This feature is similar to other fish mitochondrial genes. The complete mitogenome sequence had 16 s RNA (1,689 bp) and 12 s RNA (949 bp), which were located between tRNA-Phe and tRNA-Leu and separated by tRNA-Val gene. The location is same with most vertebrates that have high conservation.

To determine taxonomic status of *A. achilles*, we reconstructed the phylogeny of this species with other natural populations based on the *COI* gene. The phylogenetic tree showed that the *A. achilles* has the closer relationship with *Acanthurus leucostemon* (Figure 1). The phylogeny was reconstructed based on the General Time Reversible + Invariant + gamma sites (GTR + I+ G) model of nucleotide substitution using Mega7 (Kumar et al. 2016). The complete mitochondrial genome sequence of the *A. achilles* provided an important dataset for a better understanding of the mitogenomic diversities and evolution in fish as well as novel genetic markers for studying population genetics and species identification.

**Figure 1.**
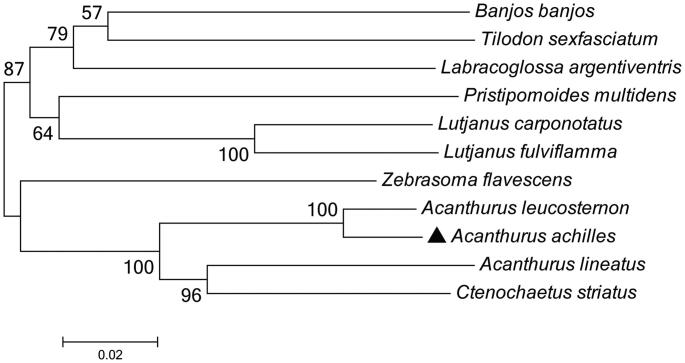
The phylogenetic relationship was estimated using the Maximum Likelihood method for the *COI* genes. Genbank accession Numbers: *Acanthurus leucosternon* (EU136032), *Acanthurus lineatus* (EU273284), *Ctenochaetus striatus* (KU244260), *Zebrasoma flavescens* (AP006032), *Labracoglossa argentiventris* (AP011062), *Banjos banjos* (KT345965), *Lutjanus fulviflamma* (NC_043916), *Tilodon sexfasciatum* (AP014538), *Pristipomoides multidens* (KF430626), *Lutjanus carponotatus* (NC_044104) and *Acanthurus achilles* (MN872231). The numbers at the nodes are bootstrap percent probability values based on 1000 replications.

## References

[CIT0001] Dierckxsens N, Mardulyn P, Smits G. 2017. NOVOPlasty: de novo assembly of organelle genomes from whole genome data. Nucleic Acids Res. 45(4):e18 28204566 10.1093/nar/gkw955 PMC5389512

[CIT0002] Kumar S, Stecher G, Tamura K. 2016. MEGA7: Molecular Evolutionary Genetics Analysis Version 7.0 for Bigger Datasets. Mol Biol Evol. 33(7):1870–1874. 27004904 10.1093/molbev/msw054 PMC8210823

[CIT0003] Randall JE. 1956. A revision of the surgeon fish genus acanthurus. Pacific Science. 10(2):159–235.

